# Association between compound PM_2.5_-cold events and population-specific mortality: a time-trend ecologic study of damage amplification in Zigong, China

**DOI:** 10.1186/s13690-026-01935-x

**Published:** 2026-05-18

**Authors:** Yizhang Xia, Wei Huang, Zheng Zhang, Yang Li, Yu Chen, Haili Ren, Fanqi Meng, Xiaopeng Qin, Peijie Jiang, Xinye Jin, Boda Zhou, Xi Chen

**Affiliations:** 1 National Key Laboratory for Infectious Disease Control and Prevention-Zigong Research Base for Emerging Infectious Disease, Zigong Center for Disease Control and Prevention, Zigong, 643000 China; 2 Beijing Tsinghua Changgung Hospital, School of Clinical Medicine, Tsinghua University, Beijing, 102218 China; 3 School of Psychology, Center for Studies of Psychological Application, Guangdong Key Laboratory of Mental Health and Cognitive Science, South China Normal University, Guangzhou, 510000 China; 4 Sichuan Provincial Center for Disease Control and Prevention, Chengdu, 610041 China; 5 School of Health and Intelligent Engineering, Chengdu Medical College, Chengdu, 610500 China; 6 School of Nursing, Nanjing Medical University, Nanjing, 211166 China; 7 The First Affiliated Hospital of Guangxi Medical University, Nanning, 530021 China; 8 Xinqiao Hospital, Army Medical University, Chongqing, 400037 China; 9 Hangzhou Center for Disease Control and Prevention (Hangzhou Institute of Health Supervision), Hangzhou, 310000 China

**Keywords:** PM_2.5_, Cold spell, Mortality, Combined exposure events, Time series

## Abstract

**Background:**

With the global increase in extreme weather events, understanding the effects of consecutive extreme PM_2.5_(EPM) and cold spells (CS) events on specific mortality is vital.

**Methods:**

Daily meteorological, air pollution, and mortality data were collected in Zigong. Using the Distributed Lag Nonlinear Model (DLNM), we defined the lag as 14 days and quantified the risk effect of EPM-CS events (P_95_ for EPM, P_7.5_ for CS) on resident mortality and explored the potential amplification of damage resulting from different patterns of sequential extreme events. Additionally, we calculated the attributable fraction (AF) of extreme events and conducted stratified analyses based on age, gender, marital status, etc.

**Results:**

Exposure to cold spells, PM_2.5_, and compound events was statistically associated with an increased risk of mortality. The cumulative rate ratios (CRRs) of EPM-CS events for total non-accidental mortality was 1.56(1.44,1.69). The mortality risks of EPM-CS events in females, elderly people ≥ 65 years, low level of education, and widowed, divorced, and never married were higher, with AF were 6.64%(95%CI: 5.42%, 7.89%),6.51%(95%CI: 5.52%, 7.51%), 6.10%(95%CI: 5.17%, 7.06%) and 7.73%(95%CI: 6.07%, 8.51%), respectively. The attributable fraction of specific mortality due to the EPM-CS events was the highest for cerebrovascular disease. Exposure to combined events was associated with a substantial increase in mortality risk, and the damaging effect of combined events occurring in the short term was more significant.

**Conclusion:**

Our findings demonstrated synergistic mortality risks from compound cold and pollution exposure, highlighting a disproportionate impact on vulnerable populations. This evidence supports the rationale for developing integrated early warning systems as a targeted intervention.

**Supplementary Information:**

The online version contains supplementary material available at 10.1186/s13690-026-01935-x.

**Table:** 

Text box 1. Contributions to the literature	
• This study shifts the focus from single environmental hazards to the analysis of combined extreme events, uncovering a synergistic effect on mortality that is often overlooked in existing public health policies. • It identifies specific high-risk populations and disease categories, particularly cerebrovascular diseases, thereby facilitating more targeted prevention strategies. • By demonstrating that shorter intervals between extreme events lead to worse health outcomes, the findings provide direct scientific support for integrating pollution and meteorological warnings into a coordinated and timely public health alert system.	

## Introduction

 In the context of global climate change, extreme environmental events are more frequent, including heat waves, cold spells, floods, droughts, and air pollution, which pose a significant threat to human health [1–4]. A comprehensive analysis of studies globally has revealed that cold weather has been connected to a considerable mortality burden worldwide over the last twenty years, with an annual excess of 3.03 deaths per 100,000 individuals attributed to cold spells [5]. Previous studies have reported that extreme weather events can result in a variety of adverse health outcomes, including injuries, fatalities, as well as respiratory and cardiovascular diseases [6–8]. Furthermore, air pollution, especially particularly fine particulate matter (PM_2.5_) has a substantial impact on the burden of circulatory and respiratory diseases [9, 10].

In the past decades, the effects of single extreme weather events have been confirmed by numerous studies, and further attention is now being paid to the effects of composite events. Studies have found that heat wave and cold wave events may accelerate breathing rates and affect ventilation, leading to an increase in pollutant inhalation [11]. Recent studies have demonstrated that heat waves and cold waves interact with PM_2.5_ pollution, leading to an elevated risk of mortality associated with circulatory disease [12–14]. Furthermore, a study indicated that co-exposure to both PM_2.5_ and cold spells at different altitudes increases the risk of Ischemic heart disease (IHD) hospitalization rates, with a higher attributable fraction attributed to high-altitude areas [15]. Another nationwide study from China reported that a higher all-cause mortality risk was associated with exposure to concurrent events than exposure to a single event, higher impact of concurrent events on circulatory and respiratory mortality [16]. These studies suggest that heat waves, cold spells, and air pollutants can increase the adverse effects on health.

Although some studies have investigated the concurrent effects of temperature and air pollution [14, 17, 18], while few studies, to our knowledge, have explored the concurrent effect of cold spells and PM_2.5_ pollution in Sichuan Basin of southeastern China. This region presents a unique setting where frequent temperature inversions and high humidity trap cold air and pollutants, exacerbating PM_2.5_ pollution during cold spells [19]. This synergy creates a distinct compound exposure scenario, yet its population health impacts remain inadequately quantified. Moreover, the frequency or occurrence interval of extreme weather events also has varying effects, with more pronounced impacts observed in the short term [20]. However, it is still uncertain whether the interval and intensity have distinct impacts on the effect of sequential EPM and CS events on population-specific mortality in basin climates. Consequently, it is essential to assess the health consequences of being exposed to both extreme cold and particulate air pollution in the Basin area.

Given the current research framework, we focused on sequential EPM-CS events, where a cold spell follows an extreme pollution episode within a set lag period (See the Methods for detailed definitions). Our primary objectives were to quantify the association of these events with mortality and to identify vulnerable subgroups. Additionally, we aimed to compare the health effects across events with varying characteristics, specifically the interval between events and their intensities. In this study, we conducted a population-based time-trend ecologic study using mortality data from over 0.12 million deaths in Zigong, southeastern China(2016–2021). This study aimed to assess the effects of exposure to co-occurring extreme cold and PM_2.5_ on total non-accidental (Total, A00-R99), respiratory diseases (RD, J00-J99), Chronic obstructive pulmonary disease (COPD, J40-J44), cardiovascular disease (CVD, I00-I99), cerebrovascular diseases (CD, I60-I69), or ischemic heart disease (IHD, I20-25). Additionally, the attributable fractions (AFs) were calculated to assess the mortality burden attributable to consecutive extreme events.

## Materials and methods

### Study area and timing

Zigong (east longitude: 102°02′–105°16′ and north latitude: 28°55′–29°38′) is located in the southeastern part of the Sichuan Basin of China, which has a typical subtropical monsoonal humid climate with the occurrence of rainy and overcast weather conditions is frequently observed. Figure S1 shows the location of Zigong city within China. In 2021, Zigong city had a permanent resident population of approximately 2.467 million, with a crude death rate and a non-accidental death rate of 6.48 and 6.09 per 1,000 population, respectively. The study period spanned from January 1, 2016, to December 31, 2021. Since cold spells and PM_2.5_ pollution mainly occur during the cold season, data from November to March each year were used for our analysis, encompassing all mortality and environmental exposure data during these months. In the time-series of daily cause-specific mortality and compound events from 2016 to 2021, we found that in Zigong, mortality and compound events are aggregated (Fig. S2).

### Mortality and meteorological data

Daily records of non-accidental deaths from 2016 to 2021 were obtained through the China Cause of Death Reporting System (CDRS), which covers all permanent residents of Zigong. Each data entry contains essential demographic features like age, gender, education level, marital status, and the date and main cause of mortality. According to the International Classification of Diseases, Tenth Revision (ICD-10), the mortality data are classified as death from total deaths (A00-R99), respiratory diseases(J00-J99), Chronic obstructive pulmonary disease(40-J44), cardiovascular disease(I00-I99), cerebrovascular diseases(I60-I69), or ischemic heart disease(20–25). A stratified analysis was ultimately conducted, categorizing participants based on gender, age groups (0–64 years and ≥ 65 years), educational level (categorized as low or high, low: junior high school or less, and high: senior high school or above), and marital status (married or alternative marriage status).

Daily meteorological data were collected from the Zigong Bureau of Meteorology. These data included daily mean temperature, relative humidity, and atmospheric pressure. Daily air pollution data including particulate matter < 2.5 μm in aerodynamic diameter (PM_2.5_, 24-h mean µg/m^3^), sulfur dioxide (SO_2_, 24-h mean µg/m^3^), nitrogen dioxide (NO_2_, 24-h mean µg/m^3^), and ozone (O_3_, daily maximum 8-hour moving average concentration µg/m^3^), were obtained from municipal environmental monitoring sites in Zigong. These data were collected from a network of eight air quality monitoring stations (four national-control and four provincial-control), ensuring comprehensive spatial coverage across all six administrative districts of the city (Figure S3 shows the location of air quality monitoring stations in Zigong). The city-wide daily average was derived as the arithmetic mean of these station-specific 24-hour averages. For ozone, the maximum 8-hour moving average concentration was used as the daily metric. For the environmental data, the overall percentage of missing data was 0.85%, and missing values were interpolated using the average of three adjacent values.

### Definition of extreme events

Currently, there is no uniform definition of extreme events, we adopted the percentile method for definition, based on previous studies [5, 20]. Previous studies have utilized minimum temperature, apparent temperature, and other metrics to define cold spells [15, 21]. However, some research has indicated that the average temperature may offer a more accurate estimation of the association between cold spells and health outcomes compared to other indicators [22]. In our study, the reference data for determining the thresholds (95th percentile for PM_2.5_ and 7.5th percentile for temperature) were derived from the full-year daily data series within the same study period for each respective variable. We described EPM as PM_2.5_ concentration ≥ its 95th percentile (P95) and CS as a minimum of 2 consecutive days with daily mean temperature ≤ its 7.5th percentile (P7.5) [13]. The PM_2.5_-cold spell event (EPM-CS) was defined as CS occurring within 14 days after the occurrence of EPM. The definition of consecutive cold spell-extreme PM_2.5_ events (CS-EPM) was similar to the above.

### Statistical analysis

#### Association of EPM-CS with deaths for cause-specific disease

Previous studies have found that the effect of extreme temperatures on deaths from different diseases is non-linear, and events such as heat waves, cold spells, and air pollution will increase the risk of disease onset or death [12, 13, 16, 23]. This study employed a time-trend ecologic design, and a time-series Poisson regression combined with DLNM was used to analyze the association between EPM-CS and population-specific mortality. To mitigate collinearity, NO_2_ was excluded from the primary model due to its high correlation (*r* > 0.6) with PM_2.5_, O_3_ and SO_2_. Finally, the model was as follows:

1 $$\begin{aligned}\:Log\left({Y}_{t}\right)&=\alpha\:+cb\left(EPM-{CS}_{t},lag\right)\\&+ns\left({Temp}_{t},df=3\right)+ns\left({RH}_{t},df=3\right)\\&+ns\left(pollutants,\mathrm{d}\mathrm{f}=3\right)+ns\left({Time}_{t},df=2\right)+DOW\end{aligned}$$

In model 1, *Y*_*t*_ represents the number of deaths from the cause-specific disease on day *t*; α is the intercept; cb(EPM-CS) is the cross-basis matrix generated by DLNM, with a maximum lag time of 14 days. In the cross-basis matrix, exposure-response was modeled with a linear integer function; the lag-response was modeled with a natural cubic spline with 3 degrees of freedom, with three internal knots placed at equally spaced values in the log scale; ns(Temp), ns(pollutants) and ns(RH) represent natural cubic spline functions with 3 degrees of freedom applied to temperature, the pollutant variable, and relative humidity, respectively; the pollutants include PM_2.5_, O_3_, and SO_2_; with natural cubic spline with an intercept and three internal knots placed at equally spaced values in the log scale. Time_t_ is the calendar date that controls the seasonal and long-term trends; DOW means the day of the week effect. Akaike’s Information Criterion (AIC) for the model was used to estimate model parameters and determine the optimal definition. In addition, we stratified the analysis by age group, gender, education level, and marital status through the above steps to explore the susceptible population. We reported the RRs and CRRs of mortality on different types of extreme events; RRs is simply the model-estimated risk at that specific lag, the CRRs was derived by summing (on the log-risk scale) the estimated lag-specific contributions across all days within the specified interval.

#### Comparing different types of extreme events

Previous studies have found that the time interval of consecutive extreme events has an effect of varying degrees [20]. In our study, we divided the EPM-CS events. The EPM-CS variable in the model (1) was changed with the following, where 0 for a non-EPM-CS day;1 for an EPM-CS event in the interval between EPM and CS was within 8–14 days; 2 for an EPM-CS event that the interval between EPM and CS was within 0–7 days. Additionally, considering the possible damage amplification effect from consecutive extreme events, a three-categorical variable was added to the model (2):

2 $$\begin{aligned}\:\:Log\left({Y}_{t}\right)&=\alpha\:+cb\left({CS}_{t},lag\right)+ns\left({Temp}_{t},df=3\right)\\&+ns\left({RH}_{t},df=3\right)+\:\:\:\:\:ns\left(pollutants,df=3\right)\\&+ns\left({Time}_{t},df=2\right)+DOW\end{aligned}$$

In model 2, CS represents different types of CS events, where CS = 0 means a non-CS day,1 for a CS day that appears alone, and 2 for an EPM-CS day(indicated a sequential type of CS). Other parameter details can be seen in the model1.

#### Attributable fraction

Within the DLNM framework, the attributable fraction was derived using the established approach for attributable risk [24]. The specific formulae are provided below:

3 $$\:b-{AF}_{x,t}=1-exp\left(-\sum\:_{l={l}_{0}}^{l}{\beta\:}_{{x}_{t-l..}l}\right)$$

In model 3, *b-AFx*,* t* represents the correlation scores attributable to the number of cases with previous exposure to *x* and time *t*, *βx*,* l* represents the sum of exposure contributions from *xt-0*,…, *xt-l*.

All statistical analyses were performed utilizing R software 4.3.3. Specifically, the “dlnm” and “splines” packages were used for analyses. A two-sided *P* < 0.05 was identified statistically significant.

#### Sensitivity analysis

The stability of the model was validated by changing the df of the model, including mean temperature, relative humidity, air pollutants (df = 3–5), and time trend (df = 2–4). Furthermore, the effect estimates of CS and EPM can vary depending on the definitions used. To test the robustness of our findings, we conducted sensitivity analyses using alternative percentile thresholds for both cold spells (CS: P_2.5_, P_5_, P_10_) and extreme PM_2.5_ events (EPM1:75 µg/m^3^, EPM2: 115 µg/m^3^. See the supplementary for detailed definitions) to determine whether the synergistic mortality effect of sequential EPM-CS events remained present.

## Results

During the cold periods from 2016 to 2021 in Zigong, the death counts were 58,661, 12,861, 4142, 23,853, 10,424, and 4989 for non-accidental, respiratory, chronic obstructive pulmonary disease, cardiovascular, cerebrovascular, and ischemic heart disease mortality, respectively and the largest number was cardiovascular diseases (Fig. S3). For the subgroup of total deaths, 24,123 were females, and 34,538 were males, age (0–64 years old: 12,222, ≥ 65 years old: 46,439), marital status (married: 35,494, others:23,167), and education level (low:55,629,high:3032), during cold periods (from November to March). The average daily mean temperature and relative humidity were 15.12 ± 4.93℃ and 77.95 ± 11.39%. The daily average concentrations were 78.92 ± 40.65 µg/m^3^ for PM_2.5_, 12.40 ± 6.12 µg/m^3^ for SO_2_ and 43.4 µg/m^3^ for O_3−8_ (Table 1). Low temperatures and high levels of PM_2.5_ pollution showed a strong correlation with non-accidental deaths (Fig. S4). Table S1 shows the results of Spearman’s correlation analysis between air pollutants and meteorological factors.

**Table 1:** Shows the descriptive statistics of air pollutants and meteorological factors in Zigong City during the cold season (from November to March), 2016 to 2021

Group	Mean ± SD	Min	*P*_25_	*P*_50_	*P*_75_	Max
Air Pollutants						
PM_2.5_(µg/m^3^)	78.92 ± 40.65	12.6	48.9	71.6	103.3	300.8
SO_2_(µg/m^3^)	12.40 ± 6.12	4.6	7.5	10.3	16.4	36.1
O_3−8 h_(µg/m^3^)	63.44 ± 28.85	13.0	40.9	58.5	78.9	165.5
Meteorological factors						
Average daily temperature/℃	15.12 ± 4.93	3.1	11.6	14.3	18.2	30.9
Average relative humidity/%	77.95 ± 11.39	34.0	71.0	80.0	86.0	100.0

O_3−8_:8-h maximum moving average concentrations. P_25_, 25th percentile; P_50_, median; P_75_, 75th percentile

### Lag effect of EPM-CS for different diseases

**Fig. 1:**
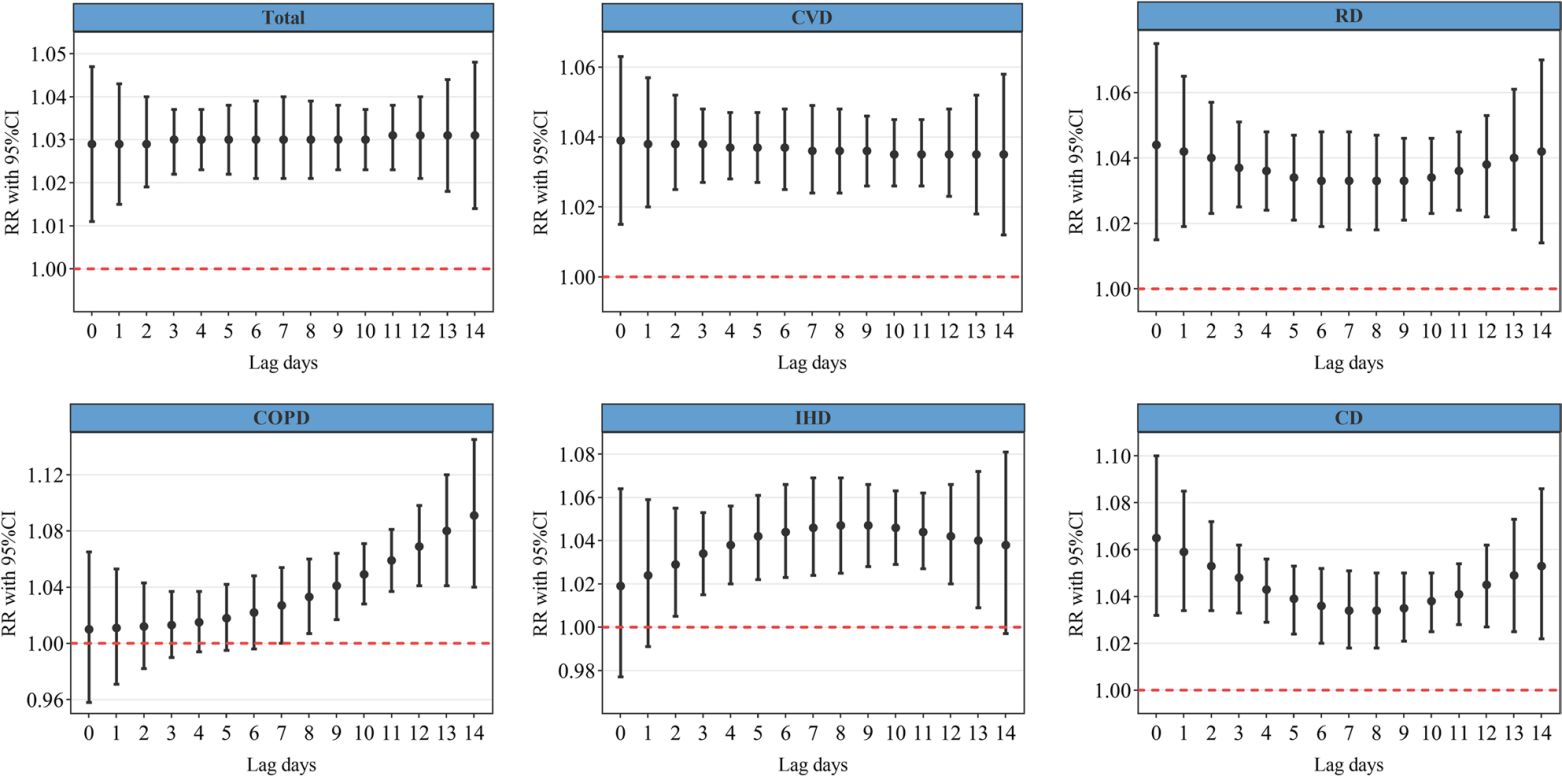
The effects of sequential EPM-CS events on specific mortality

**Fig. 2:**
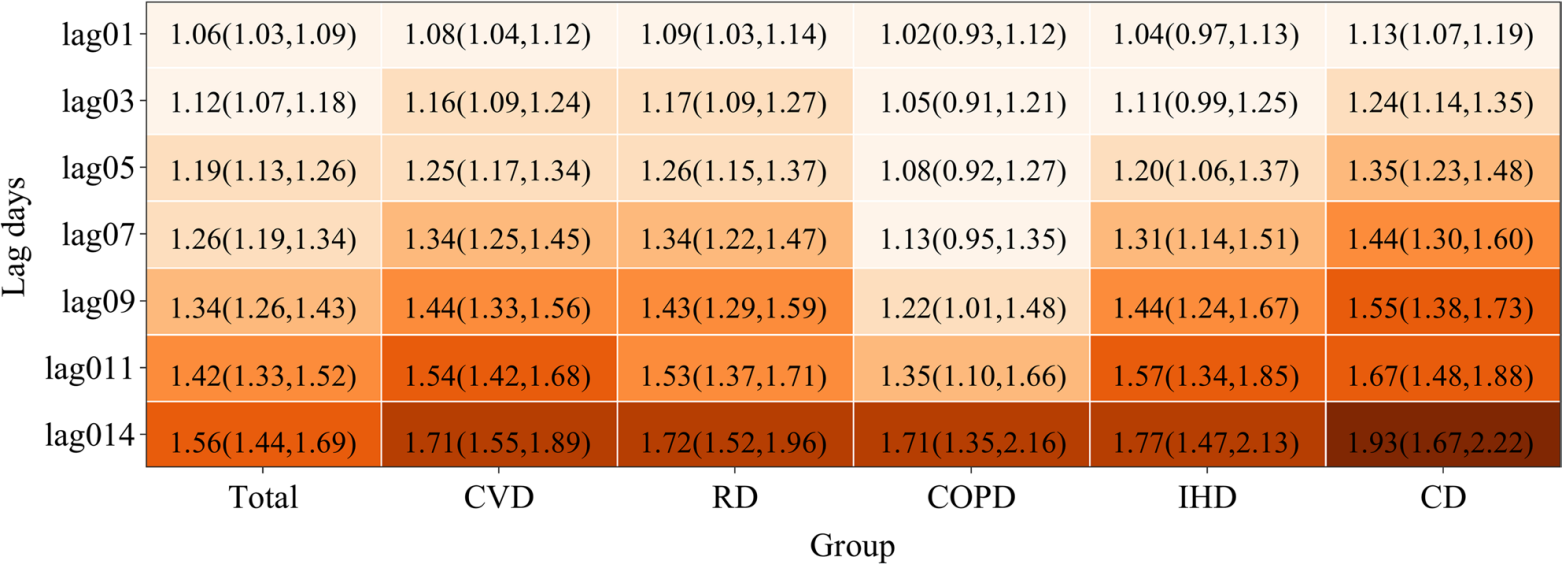
The cumulative lag effects of the EPM-CS event on population-specific mortality. Note: *RD* respiratory diseases; COPD: Chronic obstructive pulmonary disease; CVD: cardiovascular disease; CD: cerebrovascular diseases; IHD: ischemic heart disease; lag XY indicates the cumulative effect over lags X through Y (e.g., lag014 is lags 0–14)

Figures 1 and 2 show the effect of EPM-CS on different diseases in diverse lag days. The results indicate that EPM-CS was significantly associated with increased risks of non-accidental, respiratory, chronic obstructive pulmonary disease, cardiovascular, cerebrovascular, and ischemic heart disease mortality, and the confidence intervals showed a high degree ofcertainty in the estimate. The EPM-CS events related CRRs(lag014) were 1.56 (95%CI: 1.44, 1.69) for total mortality, 1.72 (95%CI: 1.52, 1.96) for RD, 1.71 (95%CI: 1.35, 2.16) for COPD, 1.71 (95%CI: 1.55, 1.89) for CVD, 1.93 (95%CI: 1.67, 2.22) for CD and 1.77 (95%CI: 1.47, 2.13) for IHD mortality. Furthermore, the lag effect of EPM-CS was statistically significant from lag0 to 14 days on CVD, RD, and CD mortality, and the risk was gradually increasing.

### The effects of EPM-CS on subgroups for total non-accidental mortality

Figure S5 and Table 2 show the results of the subgroup analyses in total non-accidental mortality. When stratified according to gender, the risk of death was statistically significant both males and females, with the risk higher for females than for males, CRR were 1.67(95%CI: 1.51,1.85),1.48(95%CI: 1.36,1.62), respectively. Within the age group, the risk of death was high for aged ≥ 65 years, with relatively narrow confidence intervals indicating good statistical precision, the CRR(lag014) was 1.62(95%CI:1.49,1.76). Moreover, subjects who had a low level of education, and of marital status other than married appeared to be more vulnerable to EPM-CS events, and the CRR(lag014) were 1.57(95% CI: 1.45,1.70),1.67(95% CI: 1.51,1.86), respectively.

**Table 2:** The single and cumulative lag effects and 95%CI of EPM-CS events on total non-accidental mortality

Lag days^*^	RR with 95%CI							
	Male	Female	0-64 years	≥65 years	married	Others ^a^	Low ^b^	High ^c^
Lag0	1.03(1.01,1.05)	1.02(1.00,1.05)	1.03(0.99,1.06)	1.03(1.01,1.05)	1.02(1.00,1.04)	1.04(1.02,1.07)	1.03(1.01,1.05)	1.03(0.97,1.10)
Lag1	1.03(1.02,1.05)	1.03(1.01,1.04)	1.03(1.00,1.05)	1.03(1.02,1.05)	1.02(1.01,1.04)	1.04(1.02,1.06)	1.03(1.02,1.04)	1.03(0.99,1.08)
Lag3	1.03(1.02,1.04)	1.03(1.02,1.04)	1.03(1.01,1.04)	1.03(1.02,1.04)	1.02(1.01,1.03)	1.04(1.03,1.05)	1.03(1.02,1.04)	1.03(1.00,1.06)
Lag7	1.03(1.02,1.04)	1.04(1.02,1.05)	1.02(1.01,1.04)	1.03(1.02,1.04)	1.03(1.02,1.04)	1.04(1.02,1.05)	1.03(1.02,1.04)	1.02(0.99,1.05)
Lag 9	1.03(1.02,1.03)	1.04(1.03,1.05)	1.02(1.00,1.03)	1.03(1.03,1.04)	1.03(1.02,1.04)	1.03(1.02,1.04)	1.03(1.02,1.04)	1.02(0.99,1.05)
Lag11	1.02(1.02,1.03)	1.04(1.03,1.05)	1.01(1.00,1.03)	1.04(1.03,1.04)	1.03(1.02,1.04)	1.03(1.02,1.04)	1.03(1.02,1.04)	1.01(0.99,1.04)
Lag14	1.02(1.00,1.04)	1.04(1.02,1.07)	1.00(0.97,1.03)	1.04(1.02,1.06)	1.03(1.01,1.05)	1.03(1.01,1.05)	1.03(1.01,1.05)	1.01(0.95,1.07)
Lag01	1.07(1.03,1.10)	1.05(1.01,1.09)	1.05(1.00,1.11)	1.06(1.03,1.10)	1.04(1.01,1.08)	1.08(1.04,1.13)	1.06(1.03,1.09)	1.07(0.96,1.19)
Lag03	1.13(1.07,1.19)	1.11(1.04,1.18)	1.10(1.02,1.20)	1.13(1.07,1.19)	1.09(1.03,1.16)	1.17(1.10,1.24)	1.12(1.07,1.18)	1.13(0.96,1.33)
Lag07	1.26(1.18,1.35)	1.27(1.18,1.37)	1.21(1.10,1.34)	1.28(1.20,1.36)	1.21(1.13,1.30)	1.35(1.25,1.45)	1.26(1.19,1.34)	1.25(1.02,1.52)
Lag09	1.33(1.23,1.42)	1.36(1.26,1.48)	1.26(1.13,1.40)	1.36(1.27,1.45)	1.28(1.19,1.38)	1.44(1.32,1.56)	1.34(1.26,1.43)	1.29(1.04,1.61)
Lag011	1.39(1.29,1.50)	1.48(1.35,1.61)	1.29(1.15,1.46)	1.46(1.36,1.56)	1.35(1.25,1.47)	1.53(1.40,1.67)	1.43(1.34,1.53)	1.33(1.06,1.68)
Lag014	1.48(1.37,1.62)	1.67(1.51,1.85)	1.32(1.15,1.51)	1.62(1.49,1.76)	1.48(1.35,1.63)	1.67(1.51,1.86)	1.57(1.45,1.70)	1.37(1.04,1.80)

^a^Others: widowed, divorced, and never married

^b^Low education level: junior high school or less

^c^High education level: senior high school or above

*lag X indicates the single-day effect at lag X (e.g., lag0 is lag 0), lag XY indicates the cumulative effect over lags X through Y (e.g., lag014 is lags 0-14)

### The effect of EPM-CS events with different characteristics

**Fig. 3:**
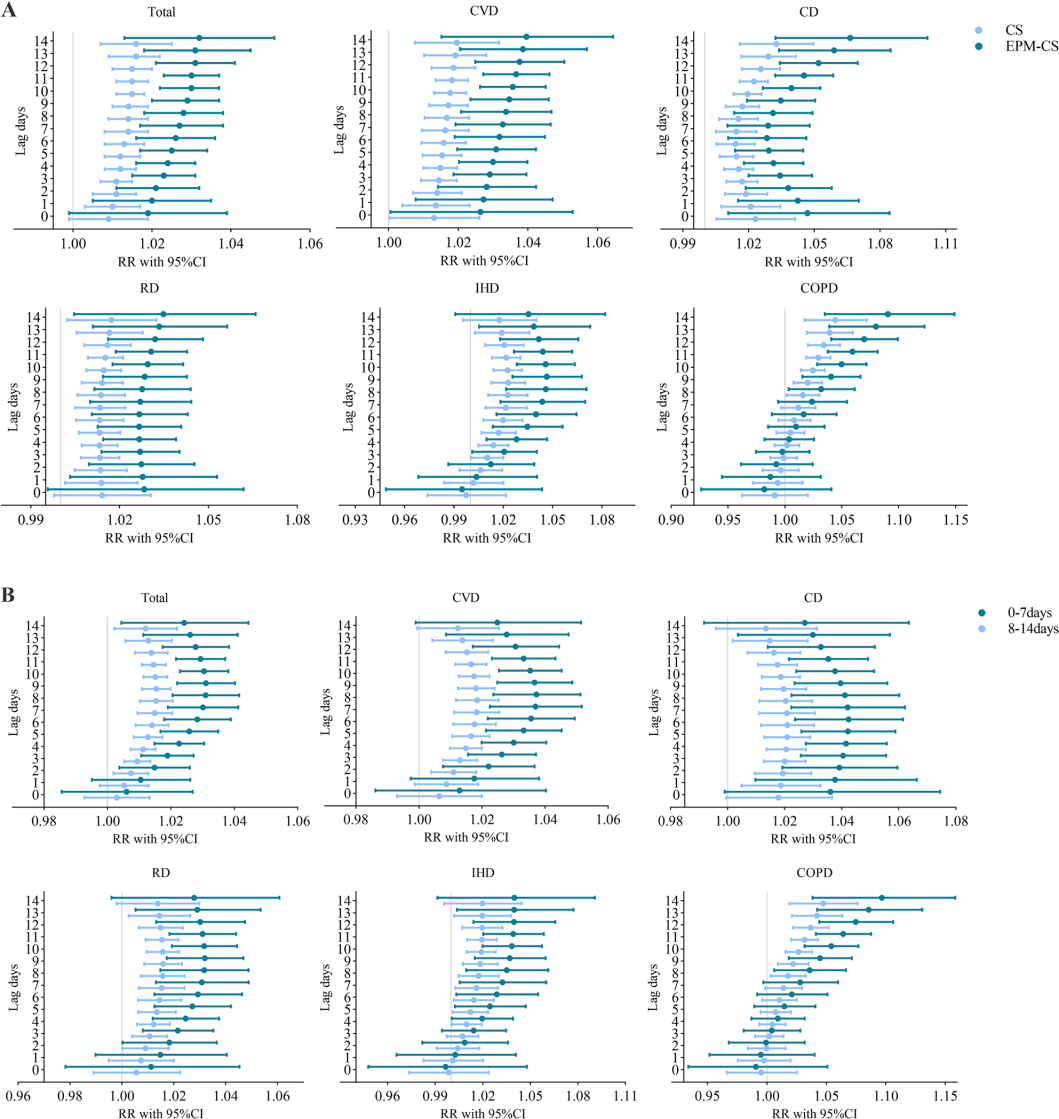
The comparison of lag effects of different types of EPM-CS events on specific mortality. **A** different patterns of extreme events. **B** different lag patterns of extreme events

Figure 3 shows the comparison of lag effects of different types of EPM-CS events on specific mortality, and we estimated the mortality risk at lag 0 to 14 days associated with CS-only and EPM-CS events (Fig. 3A). The EPM-CS events are consistently associated with a higher risk than the CS-only events. The EPM-CS events was associated with elevated risk for total non-accidental mortality (lag14RR = 1.03; 95% CI: 1.01, 1.05), CVD (lag14RR = 1.04; 95% CI: 1.02, 1.07), CD (lag14RR = 1.07; 95% CI: 1.03, 1.10), RD (lag14RR = 1.06; 95% CI: 1.01, 1.07), IHD (lag14RR = 1.04; 95% CI: 0.99, 1.08) and COPD (lag14RR = 1.09; 95% CI: 1.04, 1.15). Furthermore, our study demonstrated, for specific diseases, the overall effect of EPM-CS with the interval of 0–7 days than the event with the interval of 8–14 days (Fig. 3B). Moreover, the effect of EPM-only and CS-EPM on specific mortality can be seen in Fig. S6 and Table S2.

### The attributable fractions of EPM-CS events

**Fig. 4:**
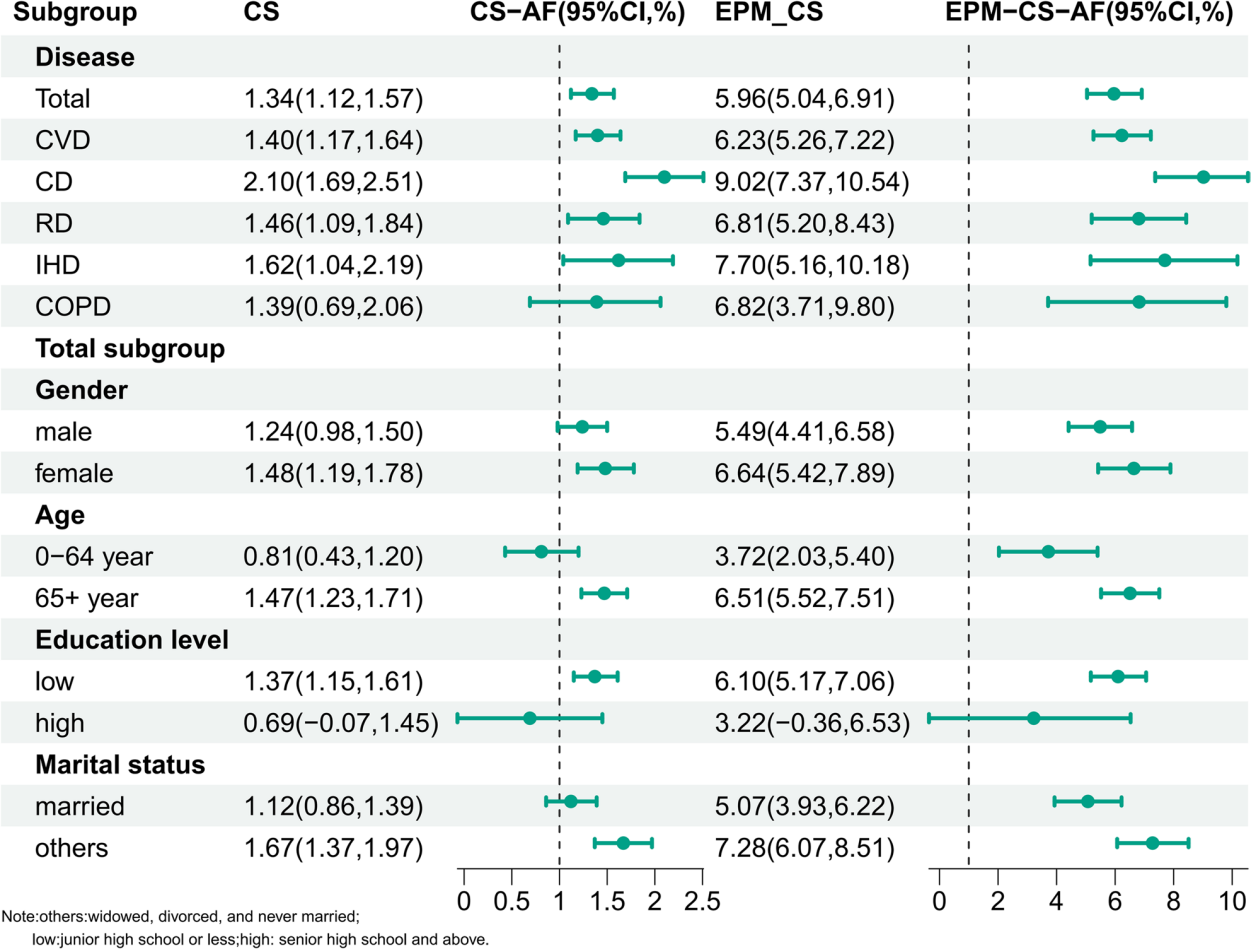
Attributable fractions of specific mortality due to different events by gender, age, educational level, and marital status

Figure 4 visualizes the levels of AFs related to EPM-CS events for different subgroups. The combined events are consistent with higher AFs than the CS-only events. In terms of specific mortality, the AF for CD was 9.02%(95%CI: 7.73%, 10.54%), which was higher than that for others. For the gender, the AF for females 6.64%(95%CI: 5.42%, 7.89%) was slightly higher than that for males 5.49%(95%CI: 4.41%, 6.58%).The elderly group ≥ 65 years of age [AF:6.51%(95%CI: 5.52%, 7.51%) for EPM-CS events, and AF:1.47%(95%CI: 1.23%, 1.71%) for CS-only events] had a higher AF for total non-accidental mortality than the age 0–64 years of age [AF:3.72%(95%CI: 2.03%, 5.40%) for EPM-CS events, and AF:0.81(95%CI: 0.43%, 1.20%) for CS-only events] for the EPM-CS and CS-only events. In terms of education level and marital status group, the people widowed, divorced, and never married had the highest AF of 7.73%(95%CI: 6.07%, 8.51%) for EPM-CS events, while the correlation of CS or EPM-CS on high education level was not significant. The attributable fractions of EPM-only and CS-EPM on specific mortality can be seen in Fig.S7. The results were generally consistent with our primary findings after varying the df (3–5) of meteorological factors, air pollutants, and the df (2–4) of time trend (Table S3 and S4). After changing the definition of CS and PM_2.5_, we found that the amplifying mortality effect associated with sequential EPM-CS events persisted across all alternative definitions (Table S5).

## Discussion

In this study, we assessed compound PM_2.5_ pollution and cold spells on population-specific mortality during the cold season from 2016 to 2021 in the Basin area and assessed the attributable burden. Our study indicates that exposure to cold spells, PM_2.5_, and combined events was significantly associated with an increased risk of mortality, and the damaging effect of combined events was more pronounced in the short term. Mortality risk associated with the combined events was more pronounced than the risk of only events for respiratory, chronic obstructive pulmonary disease, cardiovascular, cerebrovascular, and ischemic heart disease mortality. The mortality risks of a compound event in females, elderly people ≥ 65 years, low level of education, and those widowed, divorced, and never married were higher.

Globally, climate change is a serious public health challenge. The 2021 Global Burden of Disease study shows that low temperature joined particulate matter pollution as environmental risks among the top ten risk factors [25]. Over the past half-century, health risks associated with cold spells and air pollution have been increasingly documented, with the majority of studies originating from developed countries, although an increasing number of reports have emerged from developing countries in recent decades. A study covering a population of 168 million people in 209 US cities found that cold waves were associated with an increased risk of mortality, and the risk increased with the duration and intensity of cold waves [26]. Our study found that for 12 definitions of the cold spell, the effects of the cold spell increased with a lower threshold, and the effect increased with a longer duration(Fig. S8). Another study of 31 provincial capital cities in China estimated that cold waves increased the risk of respiratory diseases and COPD, with RR of 1.88 (1.65–2.11) and 1.88 (1.58–2.19) [27]. A study in Japan and Korea found that the risk and health burden of cold spells has increased over the decades, and most RRs for cold spells significantly increased during the study period in the total population, and more pronounced increases were observed in Japan-south [28]. Additionally, animal experiments found that cold stress affected their neurophysiological responses [29].

In addition, associations between exposure to PM_2.5_ and total and cause-specific mortality are well established [30–32]. PM_2.5_ might directly impact the respiratory tract by causing an imbalance in the autonomic nervous system through its interaction with lung receptors or nerves, thus enhancing susceptibility to temperature changes [33]. Therefore, this pathway may underlie the synergistic mortality effect we observed for sequential EPM-CS events. Moreover, a recent relevant epidemiological study has found that PM_2.5_ exposure may activate the gut-brain axis(GBA) by affecting gut microbiota, inflammatory factors, and so on, leading to neurological and cardiovascular system dysfunction [34]. Consequently, such mechanisms could contribute to the heightened vulnerability identified in populations with pre-existing cardiorespiratory or metabolic conditions in our study. A study covering 250 counties in China found that the risks of mortality from heavy PM_2.5_ pollution events increased by 1.14% (95% CI: 0.74–1.53%), 1.09% (95% CI: 0.58–1.60%), and 1.30% (95% CI: 0.40–2.20%), for non-accidental, circulatory, and respiratory mortality, respectively [35]. Another epidemiological time series study has reported that a 10 µg/m^3^ increment in PM_2.5_ was associated with a 1.04% (95%CI: 0.52% to 1.56%) increase in the risk of death, and associations for respiratory deaths of death were larger than for cardiovascular deaths [36]. Moreover, other studies have produced consistent results [37–39].

Although extensive research has confirmed that cold spells and high levels of PM_2.5_ pollution individually have adverse effects on hospitalization rates and mortality [26, 27, 40, 41], while little is known regarding the effects of the combined events on mortality, specifically co-exposure to PM_2.5_ pollution and cold spells. Previous studies have more attention to the combined events of heat waves and air pollution [16, 42]. Similar to previous studies, our study found that combined EPM-CS events were associated with a higher risk for cerebrovascular mortality than other mortality [16]. It is noteworthy that the greatest effect estimates of CS-EPM events were found among COPD, with AF of 9.392 (6.782,11.844). The temporal sequence of different extreme events can lead to substantially different impacts. A study in Shanghai confirmed that positive additive interactions (RERI > 0) between PM_2.5_ pollution and cold spells, especially in respiratory mortality [13].In addition, previous studies have found that the synergistic effect of cold waves and PM_2.5_ exposure varied statistically between different altitudes, and high-altitude areas exhibited a significant interaction between cold waves and PM_2.5_ [15].

Compound events exposure plausibly amplifies oxidative stress and systemic inflammation more than either exposure alone, while also promoting autonomic nervous system dysregulation that elevates cardiovascular risk. PM_2.5_ can further impair biological barriers such as the blood-brain barrier, potentially allowing particle translocation into the brain and accelerating neuroinflammation. Additionally, combined cold and PM_2.5_ exposure may enhance pro-inflammatory cytokine release, reactive oxygen species (ROS) formation, and Th1/Th2 immune imbalance, collectively intensifying integrated pathophysiological responses [43].

We also observed that females have higher risks of mortality associated with only or combined events than males, consistent with previous studies [20, 42, 44]. According to evidence from previous epidemiological studies, females seem to have less thermoregulation and lower sweating capacity, and due to physiological differences in comparison with males, and females skin temperature is more sensitive to thermal sensation than that of males under local cooling [45, 46]. However, a study in the Netherlands found that no sex differences were found in the cold [47]. Moreover, some studies also reported that the interaction between cold waves and PM_2.5_ was greater in men than in women [15]. For different age groups, the risk of death was high for aged ≥ 65 years. This could be attributed to their reduced thermoregulatory ability, existing medical conditions, weakened mechanisms for adapting to changes in ambient temperature, and the inability to access medical advice or public health services in a timely manner [48]. Moreover, we observed more attributable mortality from combined events in the low level of education, and widowed, divorced, and never married, consistent with previous studies [49], which may be explained by potential vulnerabilities in these subgroups, related to low income, limited healthcare services, poor nutrition, and working conditions [50, 51]. Similar results were found in previous studies conducted in China and Brazil [52, 53].

Our findings demonstrated synergistic mortality risks from compound cold and pollution exposure. This evidence supports the rationale for developing integrated early warning systems as a targeted intervention, pending future research on their design and effectiveness. The research found that a program aimed at delivering clean air to the most polluted areas worldwide could avoid hundreds of thousands of premature deaths every year [41]. At the same time, Chinese scholars suggest that there needs to be more emphasis on the adaptability to manage compound events amid climate change, particularly for populations at risk [16].

There are several strengths in this study. Firstly, it was the first to evaluate the impact of compound PM_2.5_ pollution and cold spells on population-specific mortality among residents in the Basin area, and the mortality data were derived from the CDRS and were therefore authentic and reliable. Additionally, we fully explored the sex, age, educational level, and marital status differences in the mortality risk for a compound event. At the same time, this study also has limitations. First, the cause of death data came from hospital records, and meteorological data from city monitoring sites rather than individual measurements, which can lead to misclassification. Second, the cause of death data, categorized using ICD-10 codes from death certificates, which may be biased. Third, residual confounding by unmeasured or imperfectly measured individual-level sociodemographic factors remains possible. While our study benefits from a complete, real-world dataset, the analysis was not preceded by a formal sample size calculation targeting a pre-specified effect size. Finally, the study area had unique geographic characteristics, climatic conditions, social, and economic status, population sizes, and other factors, so the results may not generalize to other regions.

## Conclusions

This study provided novel evidence on the increasing population-specific mortality risk and burden of co-exposure to PM_2.5_ pollution and cold spell events in the Basin area. The elderly people ≥ 65 years, females, low level of education, and widowed, divorced, and never married were particularly vulnerable during days with combined events. This disproportionate burden underscores the necessity for equity-informed interventions. Our findings provide crucial evidence for shaping public health strategy, suggesting that exposure mitigation and early warnings for compound events should be implemented as equity-centered measures, with resources and outreach prioritized for the most vulnerable groups.

## Supplementary Information

Supplementary Material 1.

## Data Availability

The datasets used in this study are available from the corresponding author upon reasonable request.

## Abbreviations

PM_2.5_: Particulate matter < 2.5 μm in aerodynamic diameter
O3−8 h: Daily maximum 8-hour moving average concentration of ozone
RH: Relative humidity
DLNM: Distributed-lag non-linear models
AIC: Akaike’s Information Criterion
AF: Attributable fraction
SD: Standard deviation
RRs: Rate ratios
CI: confidence interval
CRRs: Cumulative rate ratios
