# Supplementary material for: Association between compound PM2.5-cold events and population-specific mortality: a time-trend ecologic study of damage amplification in Zigong, China
Source: Arch Public Health. 2026 May 18;84:128. doi: 10.1186/s13690-026-01935-x (PMC13248460; doi:10.1186/s13690-026-01935-x)
Supplement: Supplementary file 1 — Supplementary Material 1. [file 13690_2026_1935_MOESM1_ESM.docx]

**Supporting information for**

**Association between compound PM_2.5_-cold events and population-specific mortality: a time-trend ecologic study of damage amplification in Zigong, China**

Yizhang Xia^a1^,Wei Huang^a1^,Zheng Zhang^c1^,Yang Li^d^,Yu Chen^e^,Haili Ren^f^,Fanqi Meng^a^,Xiaopeng Qin^g^,Peijie Jiang^h^,Xinye Jin^i^,Boda Zhou^b*^,Xi Chen^a*^

**Supplementary Figure S1-8**

**Supplementary Table S1~5**

Figure S1: Locations of Zigong city in China. QGIS 3.30.1 software was used to create the map (QGIS Development Team, 2009; QGIS Geographic Information System).

Figure S2: Time-series of daily cause-specific mortality in Zigong (2016-2021, Nov-Mar) with PM2.5 compound event periods.

Figure S3: Locations of weather and air pollution monitoring stations in Zigong. QGIS 3.30.1 software was used to create the map (QGIS Development Team, 2009; QGIS Geographic Information System).

Figure S4 : Fitting curves of non-accidental deaths to mean daily temperature and PM_2.5._

Figure S5 : The effects of sequential EPM-CS event on subgroups of total non-accidental mortality.

Figure S6 : The comparison of lag effects of different types of CS-EPM event on specific mortality.

Figure S7: Attributable fractions of specific mortality due to different events by gender, age, educational level, and marital status.

Figure S8 : Cumulative effect of lag 0-14 for total mortality under 12 definitions of cold spells.

Table S1: Spearman’s correlation analysis between air pollutants and meteorological factors.

Table S2: The single and cumulative lag effects and 95%CI of CS-EPM event on population specific mortality.

Table S3: Total sensitivity analysis of the model(time trend freedom 2-4 and meteorological factors trend freedom 3-5).

Table S4: The overall CRR of daily total mortality associated with CS and EPM-CS, with and without adjustment of air pollutants.

Table S5 Sensitivity analysis of the association between sequential EPM-CS events and mortality using alternative percentile thresholds for cold spells (P2.5, P5, P10) and extreme PM_2.5_ events (EPM1, EPM2)

**Figure S1**


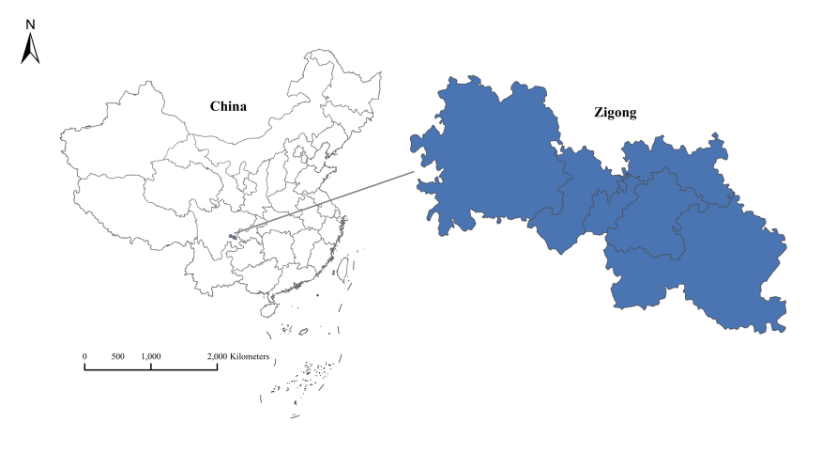


Figure S1 Locations of Zigong city in China. QGIS 3.30.1 software was used to create the map (QGIS Development Team, 2009; QGIS Geographic Information System).

**Figure S2**

Figure S2: Time-series of daily cause-specific mortality in Zigong (2016-2021, Nov-Mar) with PM2.5 compound event periods.

**Figure S3**


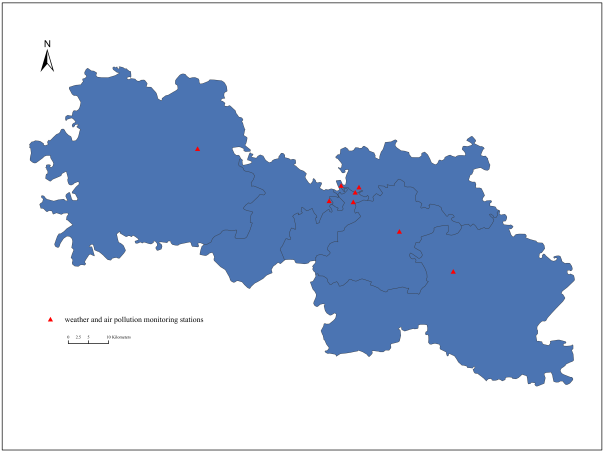


Figure S3 Locations of weather and air pollution monitoring stations in Zigong. QGIS 3.30.1 software was used to create the map (QGIS Development Team, 2009; QGIS Geographic Information System).

**Figure S4**


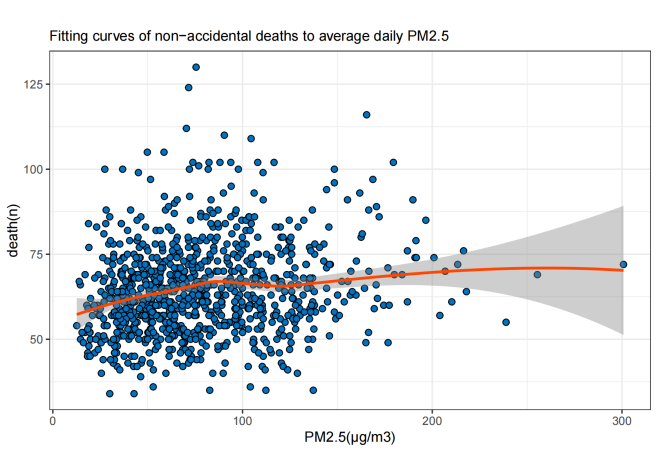

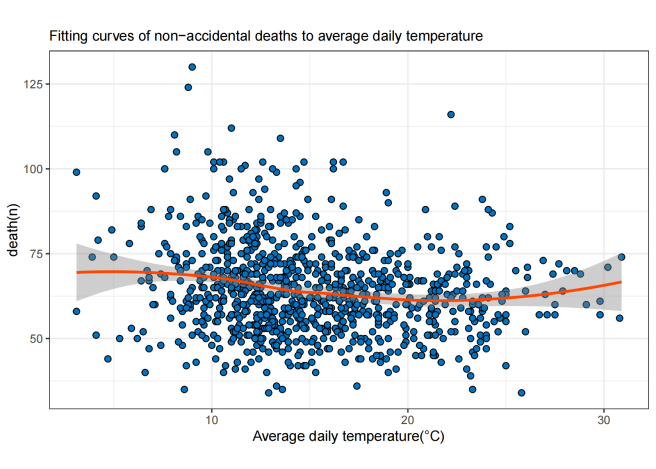


Figure S4 Fitting curves of non-accidental deaths to mean daily temperature and PM_2.5_

**Figure S5**


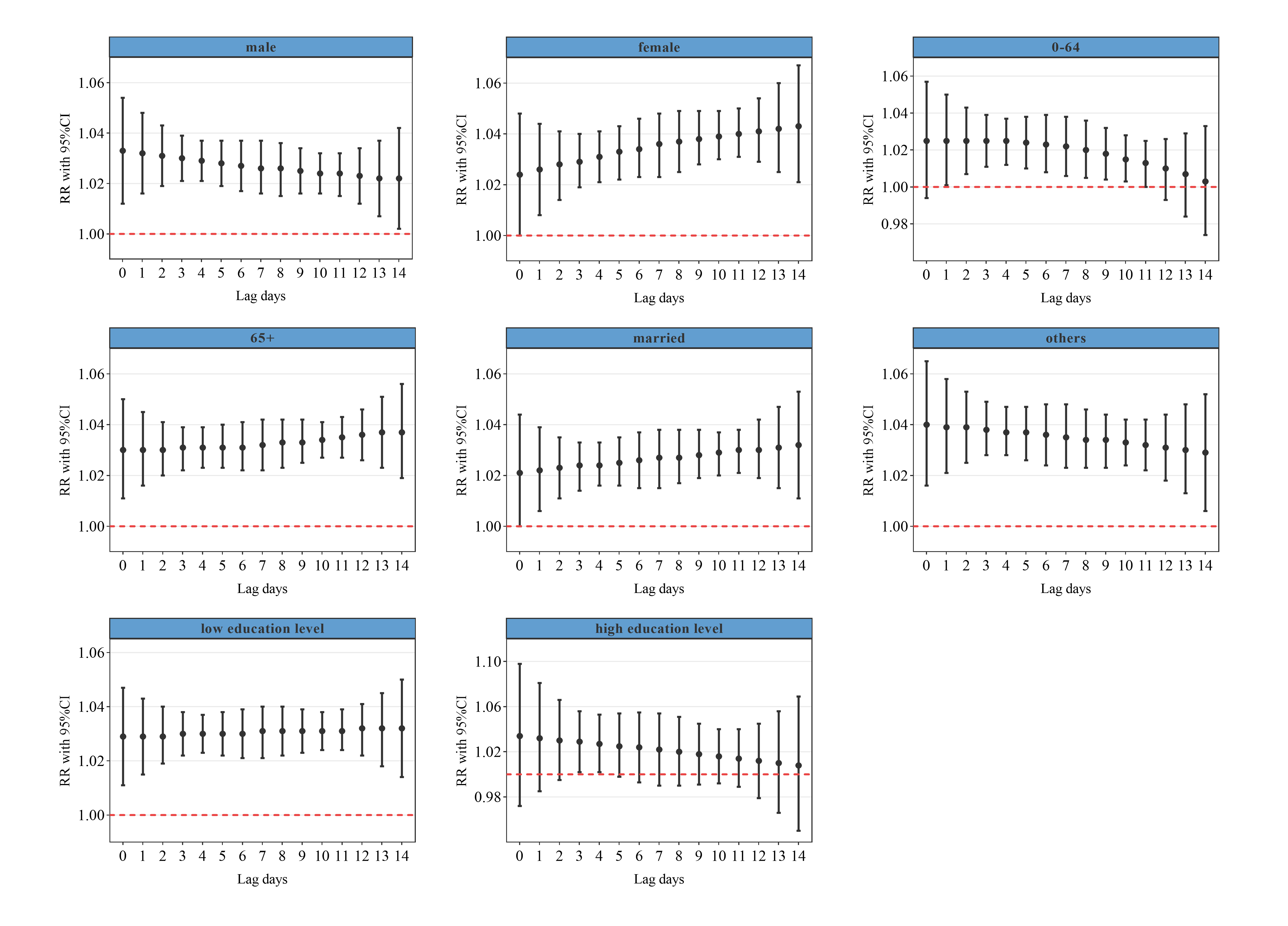


Figure S5 The effects of sequential EPM-CS event on subgroups of total non-accidental mortality.

**Figure S6**


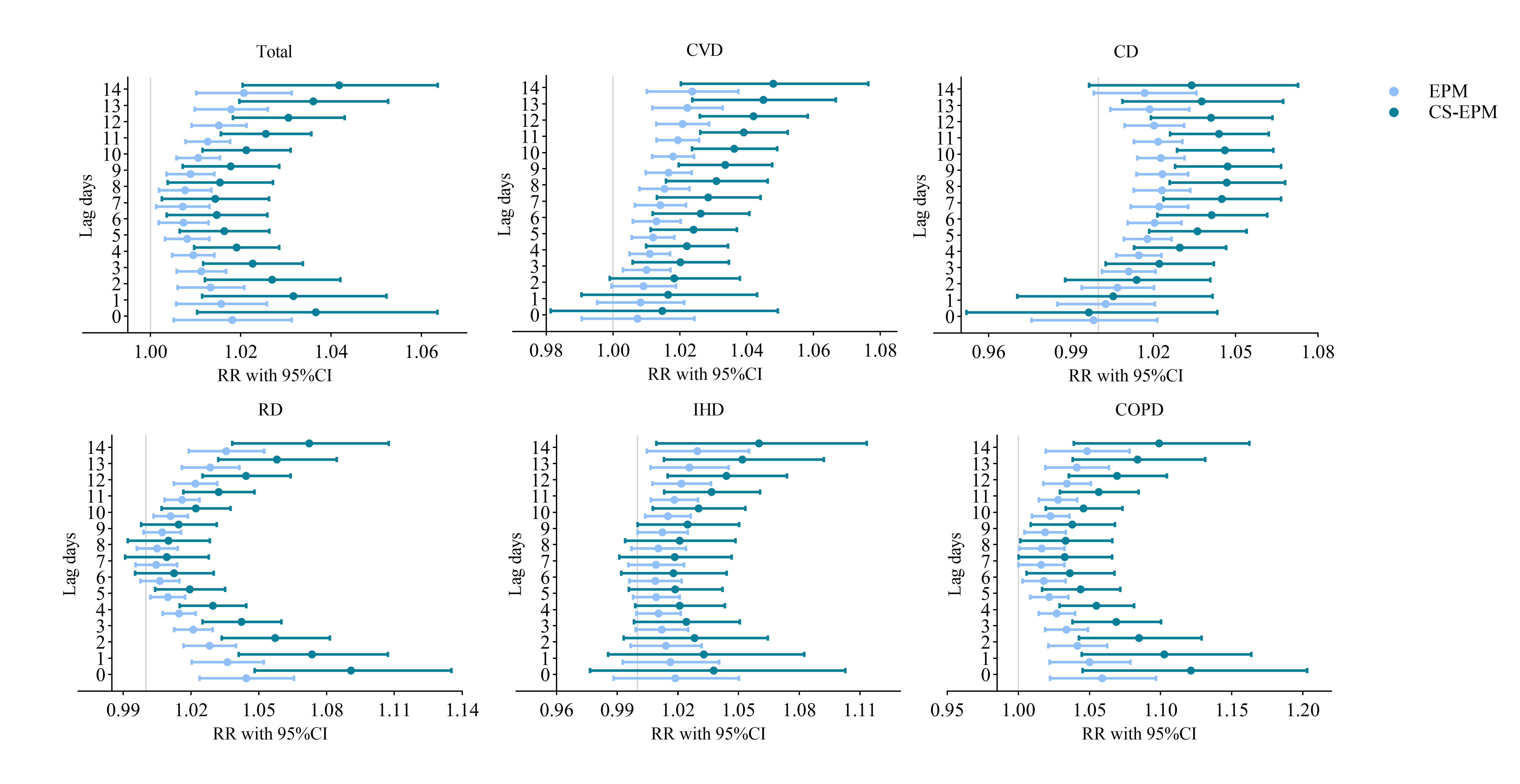


Figure S6 The comparison of lag effects of different types of CS-EPM event on specific mortality.

**Figure S7**


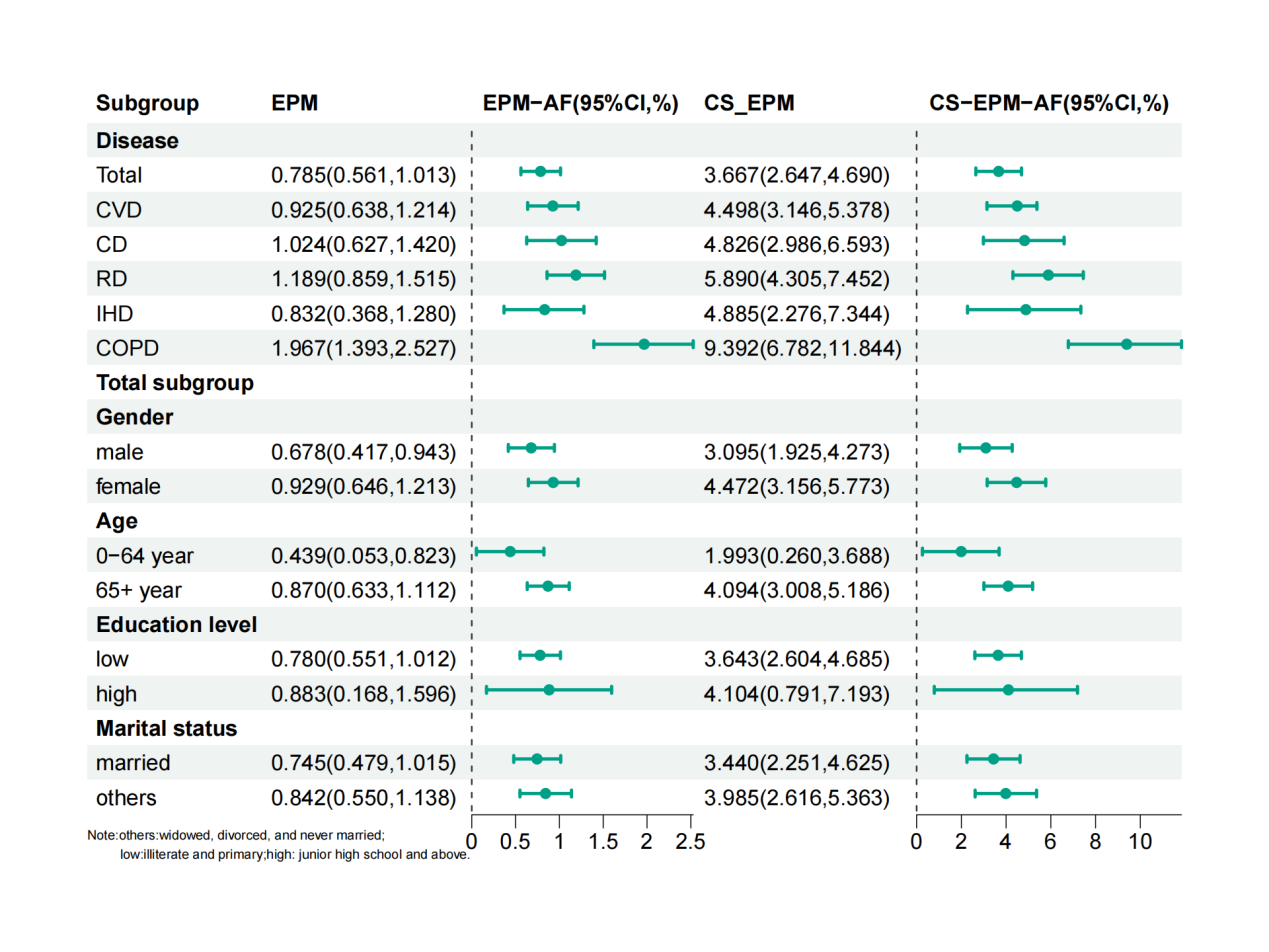


Figure S7 Attributable fractions of specific mortality due to different events by gender, age, educational level, and marital status.

**Figure S8**


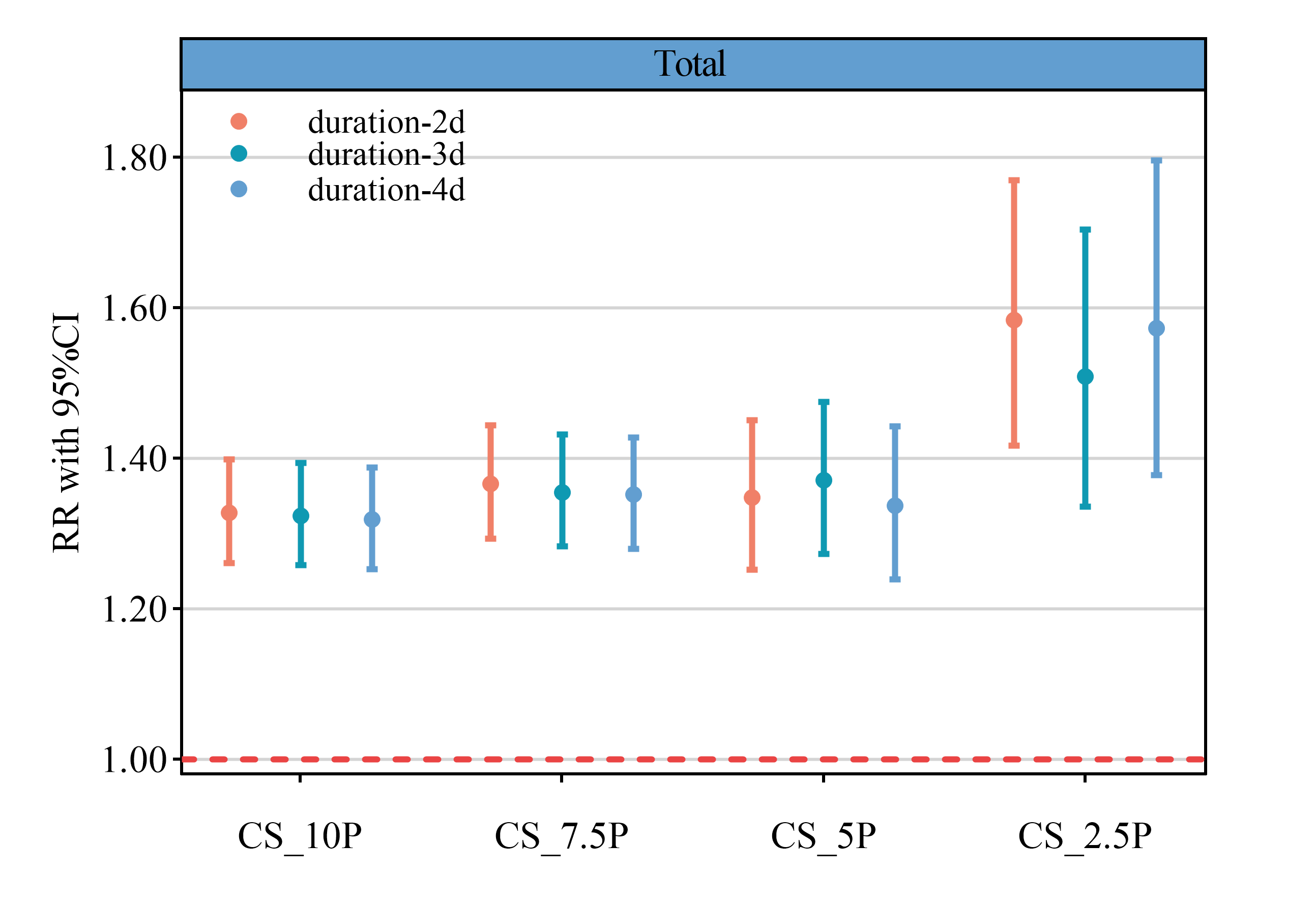


Figure S8 Cumulative effect of lag 0-14 for total mortality under 12 definitions of cold spells.

Table S1 Spearman’s correlation analysis between air pollutants and meteorological factors

| Variables | Tmean | Tmax | Tmin | RH | PM_2.5_ | PM_10_ | SO_2_ | NO_2_ | O_3-8h_ |
| --- | --- | --- | --- | --- | --- | --- | --- | --- | --- |
| Tmean | 1.000 |  |  |  |  |  |  |  |  |
| Tmax | 1.000* | 1.000 |  |  |  |  |  |  |  |
| Tmin | 0.747* | 0.747* | 1.000 |  |  |  |  |  |  |
| RH | -0.450* | -0.450* | -0.171* | 1.000 |  |  |  |  |  |
| PM_2.5_ | 0.042 | 0.042 | -0.035 | -0.071* | 1.000 |  |  |  |  |
| PM_10_ | 0.114* | 0.114* | 0.011 | -0.167* | 0.972* | 1.000 |  |  |  |
| SO_2_ | 0.049 | 0.049 | 0.001 | -0.150* | 0.582* | 0.601* | 1.000 |  |  |
| NO_2_ | -0.065* | -0.065* | -0.113* | -0.027 | 0.681* | 0.692* | 0.748* | 1.000 |  |
| O_3-8h_ | 0.726* | 0.726* | 0.308* | -0.600* | 0.084* | 0.152* | 0.183* | 0.036 | 1.000 |

Note: Tmean: Mean temperature; Tmax: Max temperature; Tmin: Min temperature; RH: relative humidity;O_3-8h_: 8-h maximum moving average concentrations.

**P*< 0.05.

Table S2 The single and cumulative lag effects and 95%CI of CS-EPM event on population specific mortality.

| Lag days | RR with 95%CI | | | | | |
| --- | --- | --- | --- | --- | --- | --- |
|  | Total | CVD | RD | IHD | COPD | CD |
| Lag0 | 1.037(1.010,1.064) | 1.015(0.981,1.049) | 1.091(1.048,1.135) | 1.038(0.976,1.103) | 1.121(1.045,1.203) | 0.997(0.952,1.043) |
| Lag1 | 1.032(1.011,1.052) | 1.017(0.991,1.043) | 1.074(1.041,1.107) | 1.033(0.985,1.082) | 1.103(1.045,1.164) | 1.005(0.970,1.042) |
| Lag2 | 1.027(1.012,1.042) | 1.018(0.999,1.038) | 1.057(1.034,1.081) | 1.028(0.993,1.064) | 1.085(1.043,1.129) | 1.014(0.988,1.041) |
| Lag3 | 1.023(1.012,1.034) | 1.020(1.006,1.035) | 1.042(1.025,1.060) | 1.024(0.998,1.051) | 1.069(1.038,1.100) | 1.022(1.003,1.042) |
| Lag4 | 1.019(1.010,1.029) | 1.022(1.010,1.034) | 1.030(1.015,1.044) | 1.021(0.999,1.043) | 1.055(1.029,1.081) | 1.030(1.013,1.047) |
| Lag5 | 1.016(1.006,1.026) | 1.024(1.011,1.037) | 1.019(1.004,1.035) | 1.019(0.996,1.042) | 1.044(1.017,1.072) | 1.036(1.019,1.054) |
| Lag6 | 1.015(1.004,1.026) | 1.026(1.012,1.041) | 1.013(0.995,1.030) | 1.018(0.992,1.044) | 1.036(1.006,1.068) | 1.041(1.021,1.062) |
| Lag7 | 1.014(1.003,1.026) | 1.029(1.013,1.044) | 1.009(0.991,1.028) | 1.018(0.991,1.047) | 1.033(1.000,1.066) | 1.045(1.024,1.067) |
| Lag8 | 1.015(1.004,1.027) | 1.031(1.016,1.046) | 1.010(0.992,1.028) | 1.021(0.994,1.049) | 1.033(1.001,1.066) | 1.047(1.026,1.068) |
| Lag9 | 1.018(1.007,1.029) | 1.034(1.020,1.048) | 1.015(0.998,1.031) | 1.025(1.000,1.050) | 1.038(1.009,1.068) | 1.047(1.028,1.067) |
| Lag10 | 1.021(1.012,1.031) | 1.036(1.024,1.049) | 1.022(1.007,1.037) | 1.030(1.008,1.053) | 1.046(1.019,1.073) | 1.046(1.029,1.064) |
| Lag11 | 1.026(1.016,1.036) | 1.039(1.026,1.052) | 1.032(1.017,1.048) | 1.037(1.013,1.061) | 1.057(1.029,1.085) | 1.044(1.026,1.062) |
| Lag12 | 1.031(1.018,1.043) | 1.042(1.026,1.058) | 1.044(1.025,1.064) | 1.044(1.015,1.074) | 1.069(1.035,1.105) | 1.041(1.019,1.063) |
| Lag13 | 1.036(1.020,1.053) | 1.045(1.024,1.067) | 1.058(1.032,1.084) | 1.052(1.013,1.092) | 1.084(1.038,1.131) | 1.038(1.009,1.067) |
| Lag14 | 1.042(1.020,1.064) | 1.048(1.020,1.076) | 1.072(1.038,1.108) | 1.060(1.009,1.113) | 1.099(1.039,1.163) | 1.034(0.997,1.073) |
| Lag01 | 1.069(1.022,1.119) | 1.032(0.972,1.095) | 1.171(1.091,1.257) | 1.072(0.962,1.194) | 1.236(1.092,1.400) | 1.002(0.924,1.087) |
| Lag02 | 1.098(1.035,1.166) | 1.050(0.972,1.135) | 1.238(1.129,1.358) | 1.102(0.957,1.269) | 1.341(1.140,1.578) | 1.016(0.913,1.130) |
| Lag03 | 1.123(1.048,1.203) | 1.072(0.979,1.173) | 1.291(1.160,1.436) | 1.129(0.958,1.329) | 1.434(1.188,1.730) | 1.038(0.918,1.175) |
| Lag04 | 1.145(1.062,1.233) | 1.095(0.994,1.207) | 1.329(1.184,1.492) | 1.152(0.966,1.375) | 1.513(1.235,1.852) | 1.069(0.936,1.222) |
| Lag05 | 1.163(1.077,1.257) | 1.122(1.014,1.241) | 1.355(1.202,1.527) | 1.174(0.977,1.410) | 1.579(1.280,1.948) | 1.108(0.965,1.272) |
| Lag06 | 1.180(1.091,1.278) | 1.151(1.038,1.277) | 1.372(1.214,1.550) | 1.194(0.990,1.441) | 1.636(1.321,2.027) | 1.154(1.002,1.329) |
| Lag07 | 1.197(1.105,1.298) | 1.184(1.065,1.316) | 1.384(1.222,1.568) | 1.216(1.005,1.472) | 1.689(1.359,2.100) | 1.206(1.044,1.392) |
| Lag08 | 1.216(1.119,1.321) | 1.221(1.096,1.360) | 1.398(1.230,1.589) | 1.242(1.022,1.510) | 1.746(1.397,2.181) | 1.262(1.090,1.462) |
| Lag09 | 1.237(1.136,1.347) | 1.262(1.129,1.410) | 1.419(1.243,1.619) | 1.273(1.041,1.556) | 1.812(1.441,2.278) | 1.322(1.137,1.537) |
| Lag010 | 1.264(1.157,1.380) | 1.307(1.166,1.466) | 1.450(1.265,1.662) | 1.311(1.066,1.613) | 1.895(1.496,2.400) | 1.382(1.184,1.615) |
| Lag011 | 1.296(1.184,1.419) | 1.359(1.208,1.528) | 1.497(1.300,1.723) | 1.359(1.098,1.683) | 2.002(1.569,2.555) | 1.443(1.230,1.693) |
| Lag012 | 1.336(1.216,1.467) | 1.416(1.253,1.599) | 1.563(1.350,1.810) | 1.419(1.137,1.771) | 2.141(1.663,2.757) | 1.502(1.273,1.773) |
| Lag013 | 1.384(1.253,1.528) | 1.479(1.301,1.682) | 1.654(1.417,1.930) | 1.493(1.182,1.885) | 2.321(1.779,3.029) | 1.559(1.310,1.856) |
| Lag014 | 1.442(1.295,1.605) | 1.550(1.349,1.782) | 1.773(1.500,2.097) | 1.583(1.228,2.040) | 2.551(1.910,3.406) | 1.612(1.334,1.948) |

Note: Total:non-accidental mortality;RD: respiratory diseases; COPD: Chronic obstructive pulmonary disease ; CVD: cardiovascular disease; CD: cerebrovascular diseases; IHD: ischemic heart disease.

Table S3 Total sensitivity analysis of the model(time trend freedom 2-4 and meteorological factors trend freedom 3-5)

| Event | Value | df | CRR and 95%CI | | |
| --- | --- | --- | --- | --- | --- |
|  |  |  | lag01 | lag07 | lag014 |
| CS-only | Time | 2 | 1.022(0.989,1.057) | 1.114(1.057,1.175) | 1.340(1.265,1.421) |
|  |  | 3 | 1.021(0.988,1.056) | 1.111(1.053,1.172) | 1.334(1.258,1.414) |
|  |  | 4 | 1.020(0.987,1.055) | 1.105(1.047,1.165) | 1.327(1.252,1.408) |
|  | Tmean | 3 | 1.022(0.989,1.057) | 1.114(1.057,1.175) | 1.340(1.265,1.421) |
|  |  | 4 | 1.021(0.987,1.055) | 1.111(1.053,1.173) | 1.338(1.262,1.418) |
|  |  | 5 | 1.021(0.988,1.056) | 1.112(1.054,1.174) | 1.339(1.263,1.420) |
|  | RH | 3 | 1.022(0.989,1.057) | 1.114(1.057,1.175) | 1.340(1.265,1.421) |
|  |  | 4 | 1.022(0.989,1.057) | 1.114(1.056,1.175) | 1.340(1.264,1.420) |
|  |  | 5 | 1.022(0.989,1.057) | 1.114(1.056,1.175) | 1.339(1.264,1.420) |
|  | Air pollutants | 3 | 1.022(0.989,1.057) | 1.114(1.057,1.175) | 1.340(1.265,1.421) |
|  |  | 4 | 1.021(0.988,1.056) | 1.113(1.055,1.174) | 1.340(1.265,1.421) |
|  |  | 5 | 1.022(0.988,1.056) | 1.111(1.054,1.172) | 1.340(1.265,1.421) |
| EPM-CS | Time | 2 | 1.059(1.027,1.093) | 1.263(1.193,1.337) | 1.559(1.442,1.685) |
|  |  | 3 | 1.060(1.027,1.093) | 1.262(1.192,1.336) | 1.553(1.436,1.678) |
|  |  | 4 | 1.058(1.026,1.092) | 1.255(1.185,1.329) | 1.538(1.422,1.663) |
|  | Tmean | 3 | 1.059(1.027,1.093) | 1.263(1.193,1.337) | 1.559(1.442,1.685) |
|  |  | 4 | 1.057(1.024,1.090) | 1.259(1.189,1.333) | 1.557(1.440,1.683) |
|  |  | 5 | 1.057(1.024,1.091) | 1.259(1.188,1.334) | 1.556(1.439,1.683) |
|  | RH | 3 | 1.059(1.027,1.093) | 1.263(1.193,1.337) | 1.559(1.442,1.685) |
|  |  | 4 | 1.059(1.027,1.093) | 1.263(1.193,1.337) | 1.558(1.441,1.685) |
|  |  | 5 | 1.059(1.027,1.093) | 1.263(1.192,1.337) | 1.557(1.440,1.684) |
|  | Air pollutants | 3 | 1.059(1.027,1.093) | 1.263(1.193,1.337) | 1.559(1.442,1.685) |
|  |  | 4 | 1.059(1.026,1.092) | 1.263(1.192,1.338) | 1.562(1.444,1.689) |
|  |  | 5 | 1.059(1.027,1.093) | 1.262(1.191,1.337) | 1.558(1.440,1.685) |

Note: CS-only:minimum of 2 consecutive days with daily mean temperature ≤ its P7.5; EPM-CS: Cold spell occurring within 14 days after the occurrence of extreme PM_2.5_ pollution; Air pollutants: PM_2.5_,SO_2_,O_3-8h_;CRR:cumulative relative risk; CI: confidence interval.

Table S4 The overall CRR of daily total mortality associated with CS and EPM-CS, with and without adjustment of air pollutants

| Event | Value | df | CRR and 95%CI | | |
| --- | --- | --- | --- | --- | --- |
|  |  |  | lag01 | lag07 | lag014 |
| CS-only | +PM_2.5_ | 3 | 1.033(0.999,1.068) | 1.133(1.075,1.194) | 1.353(1.280,1.430) |
|  | +SO_2_ | 3 | 1.031(0.998,1.065) | 1.138(1.080,1.199) | 1.389(1.316,1.467) |
|  | +O_3-8h_ | 3 | 1.025(0.992,1.059) | 1.136(1.078,1.197) | 1.362(1.289,1.439) |
|  | +PM_2.5_ | 4 | 1.033(0.999,1.067) | 1.132(1.073,1.193) | 1.352(1.279,1.429) |
|  | +SO_2_ | 4 | 1.031(0.997,1.065) | 1.138(1.080,1.199) | 1.390(1.317,1.468) |
|  | +O_3-8h_ | 4 | 1.025(0.991,1.059) | 1.136(1.078,1.198) | 1.362(1.289,1.439) |
|  | +PM_2.5_ | 5 | 1.033(0.999,1.068) | 1.132(1.074,1.193) | 1.352(1.279,1.430) |
|  | +SO_2_ | 5 | 1.031(0.998,1.066) | 1.137(1.079,1.198) | 1.391(1.318,1.468) |
|  | +O_3-8h_ | 5 | 1.025(0.992,1.060) | 1.136(1.078,1.198) | 1.361(1.289,1.438) |
|  | Without air pollutants | / | 1.032(0.998,1.067) | 1.149(1.090,1.211) | 1.390(1.317,1.466) |
| EPM-CS | +PM_2.5_ | 3 | 1.064(1.031,1.097) | 1.272(1.202,1.346) | 1.576(1.462,1.699) |
|  | +SO_2_ | 3 | 1.065(1.032,1.098) | 1.290(1.220,1.365) | 1.634(1.518,1.758) |
|  | +O_3-8h_ | 3 | 1.057(1.025,1.091) | 1.280(1.210,1.355) | 1.599(1.484,1.722) |
|  | +NO_2_ | 3 | 1.058(1.026,1.092) | 1.265(1.195,1.340) | 1.562(1.444,1.689) |
|  | +PM_2.5_ | 4 | 1.063(1.030,1.096) | 1.270(1.200,1.344) | 1.574(1.460,1.697) |
|  | +SO_2_ | 4 | 1.065(1.033,1.099) | 1.291(1.221,1.366) | 1.636(1.520,1.761) |
|  | +O_3-8h_ | 4 | 1.057(1.025,1.091) | 1.281(1.211,1.355) | 1.600(1.485,1.725) |
|  | +NO_2_ | 4 | 1.058(1.025,1.091) | 1.263(1.193,1.337) | 1.558(1.441,1.685) |
|  | +PM_2.5_ | 5 | 1.063(1.030,1.096) | 1.270(1.200,1.345) | 1.574(1.460,1.697) |
|  | +SO_2_ | 5 | 1.065(1.033,1.099) | 1.291(1.220,1.365) | 1.635(1.519,1.760) |
|  | +O_3-8h_ | 5 | 1.057(1.025,1.091) | 1.281(1.211,1.356) | 1.600(1.484,1.724) |
|  | +NO_2_ | 5 | 1.056(1.024,1.090) | 1.260(1.190,1.334) | 1.558(1.440,1.685) |
|  | Without air pollutants | / | 1.062(1.029,1.095) | 1.292(1.221,1.367) | 1.631(1.515,1.755) |

Note: CRR:cumulative relative risk; CI: confidence interval.

Table S5 Sensitivity analysis of the association between sequential EPM-CS events and mortality using alternative percentile thresholds for cold spells (P_2.5_, P_5_, P_10_) and extreme PM_2.5_ events (EPM1, EPM2)

| Event | Definition* | Overall | AIC | CRR and 95%CI |
| --- | --- | --- | --- | --- |
| EPM(P_95_)+CS | CSP10_2 | 146 | 7271.48 | 1.430(1.335,1.532) |
|  | CSP10_3 | 137 | 7257.01 | 1.416(1.327,1.511) |
|  | CSP10_4 | 123 | 7234.74 | 1.400(1.322,1.483) |
|  | CSP7.5_2 | 112 | 7192.52 | 1.559(1.442,1.685) |
|  | CSP7.5_3 | 101 | 7208.77 | 1.458(1.370,1.551) |
|  | CSP7.5_4 | 80 | 7244.41 | 1.449(1.359,1.546) |
|  | CSP5_2 | 71 | 7297.44 | 1.584(1.444,1.737) |
|  | CSP5_3 | 62 | 7289.92 | 1.608(1.466,1.764) |
|  | CSP5_4 | 51 | 7339.19 | 1.599(1.446,1.768) |
|  | CSP2.5_2 | 31 | 7329.20 | 2.035(1.769,2.341) |
|  | CSP2.5_3 | 22 | 7391.55 | 1.997(1.698,2.348) |
|  | CSP2.5_4 | 16 | 7389.49 | 2.146(1.799,2.561) |
| Main Model (CSP7.5) | | | | |
| EPMP1^a^+CS | CSP7.5_2 | 138 | 7238.51 | 1.341(1.260,1.428) |
|  | CSP7.5_3 | 135 | 7219.87 | 1.322(1.243,1.407) |
|  | CSP7.5_4 | 117 | 7242.18 | 1.326(1.245,1.413) |
| EPMP2^b^+CS | CSP7.5_2 | 128 | 7286.66 | 1.380(1.303,1.461) |
|  | CSP7.5_3 | 116 | 7309.14 | 1.391(1.316,1.471) |
|  | CSP7.5_4 | 98 | 7332.96 | 1.389(1.314,1.469) |
| EPM-CS(7d)^c^ | CSP7.5_2 | 76 | 7340.88 | 1.425(1.321,1.538) |
| EPM-CS(10d)^d^ | CSP7.5_2 | 95 | 7290.24 | 1.419(1.327,1.518) |

* For example, CSP10_2 represents a cold spell defined as daily mean temperature lower than or equal to the 10th percentile of temperature for at least 2 consecutive days.

^a^ EPMP1 was defined as 75 μg/m^3^, which was the threshold of Chinese Ambient Air Quality Standards (GB 3095-2012) for 24h average PM_2.5_ concentration.

^b^ EPMP2 was defined as 115 μg/m^3^, which was a moderate air pollution, according to the Chinese Ambient Air Quality Index (AQI) standard.

^c^ EPM-CS(7d) was defined as CS occurring within 7 days after the occurrence of EPM.

^d^ EPM-CS(10d) was defined as CS occurring within 10 days after the occurrence of EPM.
